# Use of Occult Blood Detection Cards for Real-Time PCR-Based Diagnosis of *Schistosoma Mansoni* Infection

**DOI:** 10.1371/journal.pone.0137730

**Published:** 2015-09-11

**Authors:** Mirjam Schunk, Seleshi Kebede Mekonnen, Beyene Wondafrash, Carolin Mengele, Erna Fleischmann, Karl-Heinz Herbinger, Jaco J. Verweij, Christof Geldmacher, Gisela Bretzel, Thomas Löscher, Ahmed Zeynudin

**Affiliations:** 1 Department of Infectious Diseases and Tropical Medicine (DITM), Ludwig-Maximilians University of Munich, Munich, Germany; 2 Department of Medical Laboratory Sciences and Pathology, Jimma University, Jimma, Ethiopia; 3 Bundeswehr Institute of Microbiology, Munich, Germany; 4 Laboratory of Medical Microbiology and Immunology, St. Elisabeth Hospital, Tilsburg, the Netherlands; AC Camargo Cancer Hospital, BRAZIL

## Abstract

**Background:**

In *Schistosoma mansoni* infection, diagnosis and control after treatment mainly rely on parasitological stool investigations which are laborious and have limited sensitivity. PCR methods have shown equal or superior sensitivity but preservation and storage methods limit their use in the field. Therefore, the use of occult blood detection cards (fecal cards) for easy sampling and storage of fecal samples for further PCR testing was evaluated in a pilot study.

**Methodology:**

Stool specimens were collected in a highly endemic area for *S*. *mansoni* in Ethiopia and submitted in an investigator-blinded fashion to microscopic examination by Kato-Katz thick smear as well as to real-time PCR using either fresh frozen stool samples or stool smears on fecal cards which have been stored at ambient temperature for up to ten months.

**Principal Findings:**

Out of 55 stool samples, 35 were positive by microscopy, 33 and 32 were positive by PCR of frozen samples and of fecal card samples, respectively. When microscopy was used as diagnostic “gold standard”, the sensitivity of PCR on fresh stool was 94.3% (95%-CI: 86.6; 100) and on fecal cards 91.4% (95%-CI: 82.2; 100).

**Conclusions:**

The use of fecal cards proved to be a simple and useful method for stool collection and prolonged storage prior to PCR based diagnosis of *S*. *mansoni* infection. This technique may be a valuable approach for large scale surveillance and post treatment assessments

## Introduction

Schistosomiasis is a major parasitic infection in many parts of the world. It is caused by five different human pathogenic *Schistosoma* species with *S*. *mansoni* being the most important in terms of prevalence, morbidity, mortality, and socio-economic impact [1]. The digenic flukes circulate between humans and freshwater snails as intermediate hosts. Infection occurs when cercariae—the larval forms released by the snails—penetrate human skin during contact with infested water. Depending on the species, human infection leads to urogenital (*S*. *haematobium*) or intestinal (*S*. *mansoni*, *S*. *japonicum*, *S*. *mekongi*, *S*. *intercalatum*) schistosomiasis. The disease is closely linked to poverty and is especially prevalent in poor communities without access to safe water, lack of adequate sanitation and fecal pollution of freshwater bodies [2]. According to WHO estimates at least 243 million people required treatment for schistosomiasis in 2011. However, the number of people reported to have been treated for schistosomiasis in 2011 was only 28.1 million.

WHO has recognized schistosomiasis as a neglected tropical disease and started programs focusing on preventive treatment, snail control, improved sanitation, and health education. A major focus of current control programs is to reduce the burden of disease as well as transmission through periodic, large-scale population treatment with praziquantel, the only drug suitable for mass treatment and effective against all *Schistosoma* species. However, treatment failures have been reported in recent years and it is not clear whether these are due to true resistance or to ongoing new infections [3]. For the assessment of treatment efficacy in control programs as well as for epidemiological surveillance the availability of accurate and sensitive diagnostic methods is of utmost importance.

Diagnosis and control after treatment are still based primarily on microscopic detection of *Schistosoma* eggs in stool or urine. For intestinal schistosomiasis, the Kato-Katz technique is the method most commonly used for parasitological stool examination [4]. This method is simple, yet it is time-and labor-intensive and depends on the skill of an experienced microscopist. Especially if the parasite load is low, examination of consecutive samples is required to reach an adequate sensitivity [5, 6]. Therefore, the method has its limitations for large epidemiological studies, especially when treatment efficacy has to be evaluated [7, 8].

Several immunological and molecular techniques have been developed to overcome these limitations. Various methods for detecting antibodies offer higher sensitivity and are valuable for seroepidemiological studies or for the detection of low-intensity infections such as infections in travelers. However, usually they cannot differentiate between current or previous infection and are not able to assess treatment efficacy [2, 9]. Detection of circulating adult worm or egg antigens in blood or urine allows determining active infections and different rapid diagnostic tests have been developed based on this method. Studies validating a circulating cathodic antigen (CCA) urine assay showed a good sensitivity and specificity *for S*. *mansoni* infections and test results correlating with the intensity of infection. The well attainable biological sample, the easy handling and the fast obtainable results make this method very appealing as a diagnostic tool in endemic countries. However, disadvantages to the Kato Katz technique are that definite quantitative measure of infection cannot be provided and there is no information on the presence of other helminthic infections.[10–15].

Molecular methods are now well-established diagnostic tools and various polymerase chain reaction (PCR) based assays have been developed for the detection of *Schistosoma* DNA in feces, urine and blood. The high sensitivity and specificity of this method was confirmed in several studies [16–22]. Because of the high sensitivity, operational advantages (only one sample needed in contrast to microscopy) and the potential of a high throughput with the possibility to extend examination to other helminths (using a multiplex PCR) PCR-based methods promise to become a powerful diagnostic tool for epidemiological research and monitoring of control programs [2, 23, 24]. Recently, PCR technologies were much improved and the price of equipment and consumables has been reduced. As a consequence the technique has become integrated not only in research but also in clinical diagnostics and an increasing number of laboratories even in low-income countries have PCR technology available by now. However, in contrast to microscopy, the PCR methods usually need to be performed in well-equipped centralized facilities which may be far away from the sampling site. Therefore, an easy and practical method of sample preservation is essential for the field applicability of PCR and a premise needed for this method to perform in epidemiological studies. To overcome this problem, for a number of other infectious diseases the preservation of clinical samples (blood, salvia, feces) on filter paper for subsequent molecular testing has proved to be a reliable means to collect and store DNA of various pathogens [25–27]. This method is well established in malaria research [28]. In a study conducted by Grimes et al. this collecting method was also successfully applied using occult blood detection cards for the detection of diarrheagenic bacterial pathogens [29]. Filter paper specimens are advantageous to conventional fresh stool specimens, because they require no immediate processing and refrigeration, are stable for months and therefore very easy to transport and store. In the study of Grimes et al. [29] the use of commercially available Hemoccult Cards proved to be a convenient and economical means of collecting and transporting fecal material. In order to obtain optimal field applicability we investigated in a pilot study the use of Hemoccult cards for sampling and storage of stool samples to streamline detection of *S*. *mansoni* DNA by PCR.

## Methods

### Stool samples

Stool specimens were collected within a community survey from individuals living in the recently identified high prevalence community of Sahe Bontu village in Mana Wereda District, Ethiopia.

### Preparation and parasitological examination of stool specimens

After collection the stool specimens were transported on ice in cool boxes to JUH diagnostic laboratory the same day and coded aliquots for further molecular analyses were taken in an investigator-blinded fashion. Within 48 h after collection all stool samples were investigated for ova and parasites by a standard Kato-Katz method [30] in duplicate. Each slide was read for 10 min to 20 min independently by two experienced microscopists before being considered negative. Aliquots (400–500 mg) from fresh stool samples were frozen and stored in a 2 ml tube at -20°C. Another aliquot was smeared onto a Hemoccult™ card (Care diagnostica™, Möllersdorf, Austria), air dried and kept in plastic boxes at ambient temperature. No developer was applied. The aliquots were shipped (frozen samples on dry ice because of logistical reasons) to DITM, Munich, Germany, where molecular analysis was performed 10 months later.

### DNA extraction and real-time PCR

Genomic DNA from approximately 180–220 mg of each fresh frozen stool sample was extracted using the QIAamp Stool Kit according to the manufacturer’s suggestion (Qiagen, Hilden, Germany). For the DNA preparation of the fecal cards, disks the size of 1/3 of a single field (approx. 55 mm2) were cut out with a scalpel and placed in a 1.5 ml microcentrifuge tube. 1,4 ml ASL Buffer was added, vortexed thoroughly and incubated for 5 minutes at 95°C. The samples were centrifugated and only supernatant was used for next steps. DNA then was extracted using the QIAamp Stool Kit according to the manufacturer’s suggestion (Qiagen, Hilden, Germany). DNA was eluted with 50μl Buffer AE. Amplification and detection of *Schistosoma* DNA was performed using primers and probes targeting the internal transcribed spacer 2 (ITS2) sequence of *S*. *haematobium*, *S*. *mansoni* and *S*. *intercalatum* and internal control as described previously [7]. The amplification of each DNA sample was performed in a final volume of 25μl with PCR buffer (HotstarTaq master mix, QIAgen, Germany), 5 mM MgCl2, 5 pmol of each *Schistosoma* primer and 3.75 pmol of each PhHV-1-specific primer, 1.25 pmol of the *Schistosoma*-specific probe and PhHV-1-specific double-labelled probe and 5 μl of the DNA sample. Amplification consisted of 15 min at 95°C followed by 50 cycles of 15s at 95°C, 30s at 60°C, and 30 s at 72°C. Amplification, detection and analysis were performed with the CFX real-time detection system (Bio-Rad Laboratories). Negative and positive control samples were included in each amplification run. A test was positive when the threshold was attained within 45 PCR cycles (cycle-threshold [Ct] values < 45).

### Data analysis

Baseline data of the patients and their laboratory results (microscopy and real-time PCR) on *S*. *mansoni* were stored in a Microsoft Excel database (Microsoft, Redmond, WA, USA) (S1 Table) and later transferred to Stata software, version 9.0 (Stata Corporation, College Station, TX, USA), for statistical analysis. The sensitivity of the PCR that was performed using fecal cards and the PCR that was performed directly on stool samples was calculated, with the results of microscopy taken as “gold standard”. An approximative test (McNemar chi-square test for matched pairs of samples with categorical test results) and estimation of standard error of proportion (to calculate 95 percent confidence intervals [95%-CI] of categorical test results) were conducted to determine concordance. Significant differences were defined as p values below 0.05 or as not overlapping of 95%-CI of proportions. Ct-values of the different methods were compared using the Wilcoxon matched-pairs signed rank test.

### Validating protocol and assessing sensitivity

The analytical sensitivity was determined as lower limit of detection (LOD, lowest template concentration) for qPCR using 10-fold serial dilutions of cloned IST-2 templates (GenExpress, Berlin, Germany) from 10^7^ to 10^1^ copies performed twice on different days. The lower detection limit of the qPCR for stool vs. fecal card samples for different egg counts was determined by using serial saline-dilutions of one stool sample containing 408 eggs per gram of feces (determined by Kato-Katz stool examination). A suspension containing 260 eggs/ml was prepared and 4-fold dilutions were prepared with estimated concentrations of 65 eggs/ml, 16 eggs/ml, 4 eggs/ml, 1 egg/ml and 0.25 egg/ml and stored at -20°C until use. Subsequently 100μl of the specific dilutions were either used directly for DNA extraction as described above representing the stool sample while another 100μl were applied on fecal cards respectively. Fecal cards were then prepared for DNA extraction using only 1/3 of a single field as described above. The experiments were reproduced twice on different days.

### Ethics Statement

Ethical board approval was received by the ethical committees of Jimma University (RPGC/284/2012) and Zonal Health Bureau before the start of the survey. The objective of the study was explained to the community and informed consent sheets with detailed information of the study in local language were distributed. Prior to enrolment, written informed consent was obtained from each individual or, in case of children, their parents or legal guardians according to the requirements of the ethical committees. Anthelmintic treatment was provided free of charge based on the results of microscopy as obtained in Jimma University hospital (JUH) diagnostic laboratory.

## Results

The PCR assay was shown to be highly sensitive with a LOD of ≤10^1^ (S2 Table). The lower detection limit of the qPCR for stool vs. fecal card samples in serial stool dilutions either extracted directly or extracted after application on fecal cards was determined next. In the samples of directly extracted stool dilutions the limit of detection of *Schistosoma mansoni* DNA was equivalent to 4 eggs– 1 egg/ml. In the samples examining the stool dilutions applied on fecal cards the limit of detection was equivalent to 64 eggs/ml (Table 1).

**Table 1:** Results of qPCR for fecal card samples vs. stool/dilution samples for different egg counts.

Stool/saline dilutions	PCR fecal card sample—Ct values^#^	PCR stool/dilution sample—Ct values^#^
408 EPG	33.57/33.25	28.80/28.56
260 eggs/ml	39.57/39.19	24.10/23.87
65 eggs/ml	39.54/39.43	29.75/29.68
16 eggs/ml	-	34.83/34.91
4 eggs/ml	-	36.05/36.08
1 eggs/ml	-	-/41.41
0.25 eggs/ml	-	-

^#^ Ct-values of different days

Stool specimens from 55 individuals could be investigated by all methods. The study group was composed of 23 females and 32 males with an age range of 4 to 80 years (median age = 11 years). Microscopic stool examination by the Kato-Katz method showed eggs of *S*. *mansoni* in 35 of 55 samples (63.6%), 27 single *S*. *mansoni*-infections and 8 multiple infections (Table 2). In 12 of the 20 samples negative for *S*. *mansoni* single or multiple infections with other intestinal parasites were detected (Table 2).

**Table 2:** Parasitological examination for intestinal parasites.

*Organism*	Microscopy
***no parasite***	**8**
***single Infection/Schistosoma mansoni***	**27**
***single Infection/other intestinal parasites***	**9**
Ascaris lumbricoides	5
Hymenolepsis nana	2
Taenia spp.	1
Hookworm	1
***multiple infections/Schistosoma mansoni***	**8**
Schistosoma mansoni+ Ascaris lumbricoides	3
Schistosoma mansoni+ Hookworm	2
Schistosoma mansoni+ Trichuris trichiuris	1
Schistosoma mansoni+ Hymenolepsis nana	1
Schistosoma mansoni+ Hymenolepsis diminata+ Ascaris lumbricoides+ Trichuris trichiuris	1
***multiple infections/other intestinal parasites***	**3**
Ascaris lumbricoides+ Trichuris trichiuris	1
Ascaris lumbricoides+ Taenia spp.	1
Ascaris lumbricoides+ Hookworm	1
**Total**	**55**

Real-time PCR using fresh frozen stool aliquots and fecal card samples detected *Schistosoma*-specific DNA in 33 and 32 of 55 samples respectively (Table 3). The discordant samples were retested and discordant result was confirmed in all cases. All samples which were negative by microscopy were negative by PCR. Viral DNA from the internal control was amplified in all samples investigated.

**Table 3:** Comparison of PCR detection rates in DNA extracted from fecal cards and fresh frozen stool samples.

No. and (%)			
Subjects	Investigated	PCR-positive (fecal card)	PCR-positive(fresh stool sample)
**Controls (negative by microscopy)**	20	0 (0%)	0 (0%)
**Cases(positive by microscopy)**	35	32 (91.4%)	33 (94.3%)

**Fig 1:**
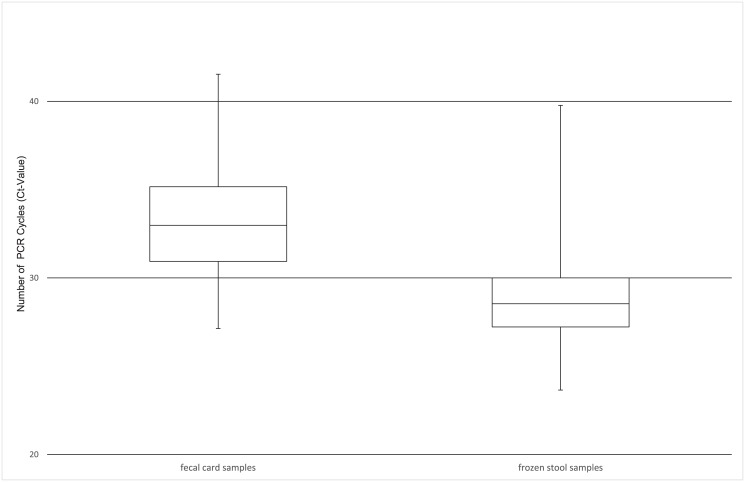
Boxplot of 30 paired PCR-positive samples comparing the distribution of Ct-values in fecal card samples (median Ct-value 33.0) and frozen stool samples (median Ct-value 28.5)

Compared to microscopy the diagnostic sensitivity of PCR was 94.3% [95%-CI: 86.6; 100] for fresh frozen stool samples and 91.4% [95%-CI: 82.2; 100] for fecal card samples. The difference between the PCR detection rates of the two sampling methods was not significant (p = 1; Fisher exact) (Table 2). The Ct values were significantly higher in the fecal card samples (median 33.0, minimum 27.1, maximum 41.5) compared to the frozen stool samples from the same individuals (median 28.5, minimum 23.6, maximum 39.8, p< 0.0001) (Fig 1).

## Discussion

In colorectal cancer screening programs fecal cards have been widely used for guaiac-based detection of occult blood in stool samples. The card method proved to be an easy and well accepted means of sample collection which can even be performed by the person tested. In a previous study Hemoccult™ detection cards have been applied for the collection of stool samples and used effectively for PCR detection of bacterial fecal DNA from various enteropathogenic *E*. *coli* [29]. We investigated the field applicability of such an easy method to collect and store stool samples for highly sensitive PCR techniques in the diagnosis of schistosomiasis in a pilot study and found a 91% sensitivity of PCR using dry fecal spots collected on commercially available Hemoccult™ cards when compared to the Kato-Katz thick smear microscopy in individuals with *S*. *mansoni* infection. *Schistosoma*-DNA could be extracted from fecal cards, stored dry at ambient temperature for up to 10 months with no statistically significant difference in comparison to stool aliquots freshly frozen, and stored at –20°C until extraction. However, Ct values were significantly higher in the fecal card samples (median 33.0) compared to the frozen stool samples (median 28.5, p< 0.0001), reflecting a lower parasite specific DNA load than in the fresh frozen stool samples. Because the sample volume that was incorporated into the extraction process was roughly one third in filter paper samples than in conventional stool samples, higher stool amounts could be smeared onto the fecal cards or more material of the fecal cards could be incorporated for DNA preparation to account for this. This limitation is also displayed in the lower sensitivity of the PCR in detecting *S*. *mansoni* DNA in diluted stool samples extracted from fecal cards in comparison to directly extracted dilution samples. As known from dried blood spots on filter paper other factors can influence the sensitivity of detection, e.g. temperature of storage, use of different filter papers and extraction methods [31, 32]. While Grimes et al. used developer arguing that this might help to stabilize DNA, we did not apply any in our study. However, the effect of developer use on the quality of DNA extracted from Hemoccult™ cards in long-term storage still needs to be evaluated.

Although microscopic examination was carried out by the Kato-Katz method for all stool samples, results were only recorded to be negative or positive with no data on the egg per gram count. Our study population lives in an endemic area and did not receive anthelmintic treatment in recent years. With high prevalence of *S*. *mansoni* infections detected in a single microscopic examination the intensity of infection can be assumed to be high. While microscopy works well in infections with high worm burden, the sensitivity of this method decreases with lower intensity of infection [33]. Lin et al. demonstrated in a study on *S*. *japonicum* infections, that examination of a single thick stool smear is enough to detect cases with moderate intensity infections with a fecal content of 250 eggs per gram (EPG) or above, while examination of six thick smears were needed to detect cases with a very low egg count per gram (below 10) [5]. PCR based methods are valued to be highly sensitive and specific. For intestinal schistosomiasis Pontes et al. developed a PCR protocol based on primers first described by Hamburger et al. [16, 34]. Testing dilutions of artificially prepared positive fecal samples, the PCR proved to be 10 times more sensitive than the Kato-Katz examination, with the PCR being able to detect DNA in fecal samples containing only 2.4 eggs per gram. This superiority in sensitivity achieved by PCR in comparison to conventional parasitological examination has been underlined in further studies using different PCR protocols [19, 22, 35]. Because of these promising results, PCR might become the “gold” standard in future Schistosomiasis diagnostics [2]. In the presented work we followed a multiplex real-time PCR-protocol detecting *Schistosoma* DNA described by Obeng et al. [7]. In the evaluation of this protocol, discrepancies in detecting *S*. *mansoni* only occurred both ways between the results of microscopy and PCR in subjects with very low egg count (defined as 15–100 EPG). In our study both—the PCR of fresh stool as well as PCR on fecal cards—showed high sensitivity (>90%) and maximum specificity (100%) with no cross reactivity to other helminthic infections. However, examining serial dilutions with defined egg-content indicated that the performance of PCR on fecal cards is less sensitive than the conventional sampling method in very light infections. Only few egg-positive cases were found negative in either the fresh-stool-specimen- or fecal-card-PCR. Possibly these cases represent very light infections, where variation in egg output and inhomogeneous distribution in feces may lead to DNA levels below the detection limit in the PCR template. As mentioned above, one limitation of the filter paper sampling method is the small sample volume, which might lead to a higher rate of false negative results in light infections.

In conclusion, the results of this study indicate that specimens of dry fecal spots collected on Hemoccult™ cards can be employed as source of DNA for the detection of *Schistosoma mansoni* with good diagnostic results even after prolonged storage at room temperature. Being particularly suitable in remote areas with tropical climate, where collection of consecutive stool samples, transport and storage conditions are often not optimal, this technique may represent a valuable approach to optimize field applicability of PCR-based diagnosis of schistosomiasis and potentially other helminth infections as well making it applicable for large scale surveillance and post treatment assessments. Further research is needed to optimize the sensitivity of fecal cards in very light infections and to test the performance under different storage conditions and storage times.

## Supporting Information

S1 Table Baseline data clinical samples. (PDF)

S2 Table Dilutions of cloned IST-2 template. (PDF)
