# Supplementary material for: Use of Occult Blood Detection Cards for Real-Time PCR-Based Diagnosis of Schistosoma Mansoni Infection
Source: PLoS One. 2015 Sep 11;10(9):e0137730. doi: 10.1371/journal.pone.0137730 (PMC4567332; doi:10.1371/journal.pone.0137730)
Supplement: S1 Table — (PDF) [file pone.0137730.s001.pdf]

S1 Table Baseline data clinical samples:

**Probe-N°**	**Sex**	**Age**	**Microscopy Schistosomiasis**	**Microscopy other parasites**	**Ct-value - fecal card**	**Ct-value - frozen stool**
4	f	5	negative		0,00	0,00
13	f	6	negative		0,00	0,00
16	f	19	negative		0,00	0,00
21	f	35	negative		0,00	0,00
32	m	9	negative		0,00	0,00
36	m	5	negative		0,00	0,00
37	m	4	negative		0,00	0,00
97	m	20	negative		0,00	0,00
11	m	4	negative	*Ascaris lumbricoides*	0,00	0,00
14	f	14	negative	*Ascaris lumbricoides*	0,00	0,00
20	m	11	negative	*Ascaris lumbricoides*	0,00	0,00
30	m	6	negative	*Ascaris lumbricoides*	0,00	0,00
40	m	5	negative	*Ascaris lumbricoides*	0,00	0,00
12	f	12	negative	*Ascaris lumbricoides + Hookworm*	0,00	0,00
39	f	25	negative	*Ascaris lumbricoides + T. trichiuris*	0,00	0,00
26	f	10	negative	*Ascaris lumbricoides + Taenia spp.*	0,00	0,00
25	f	8	negative	*H.nana*	0,00	0,00
29	m	4	negative	*H.nana*	0,00	0,00
38	m	4	negative	*Hookworm*	0,00	0,00
35	f	40	negative	*Taenia spp.*	0,00	0,00
28	f	7	*S. mansoni*	*H. nana*	38,03	0,00
94	m	11	*S. mansoni*		38,91	0,00
1	m	50	*S. mansoni*		0,00	29,17
79	m	4	*S. mansoni*		0,00	26,14
91	m	11	*S. mansoni*		0,00	30,19
95	f	10	*S. mansoni*		31,51	23,65
92	m	13	*S. mansoni*		29,67	25,83
34	f	25	*S. mansoni*		31,66	25,97
65	m	12	*S. mansoni*		34,07	26,00
33	m	7	*S. mansoni*		34,02	26,85
27	m	6	*S. mansoni*		27,13	27,14
31	f	80	*S. mansoni*		30,38	27,49
72	m	12	*S. mansoni*		35,17	27,97
88	f	15	*S. mansoni*		36,76	28,16
89	f	9	*S. mansoni*		32,01	28,24

S1 Table Baseline data clinical samples:

9	m	9	*S. mansoni*		29,97	28,49
22	f	5	*S. mansoni*		31,78	28,59
10	m	11	*S. mansoni*		41,54	29,10
50	m	14	*S. mansoni*		34,20	29,13
7	m	12	*S. mansoni*		29,39	29,26
87	m	8	*S. mansoni*		30,58	29,80
90	m	9	*S. mansoni*		35,38	29,84
81	m	8	*S. mansoni*		33,52	30,06
17	f	20	*S. mansoni*		31,08	30,35
19	m	10	*S. mansoni*		35,43	31,75
8	m	12	*S. mansoni*		34,85	32,41
6	m	11	*S. mansoni*		30,22	32,76
48	f	11	*S. mansoni*		30,89	39,77
15	f	8	*S. mansoni*	*Ascaris lumbricoides*	32,45	24,40
23	f	12	*S. mansoni*	*Ascaris lumbricoides*	35,59	25,75
70	f	11	*S. mansoni*	*Ascaris lumbricoides*	36,09	28,88
24	f	7	*S. mansoni*	*H. diminata + Ascaris lumbricoides+ T. trichiuris*	35,75	28,06
5	m	13	*S. mansoni*	*Hookworm*	35,17	28,27
93	m	36	*S. mansoni*	*Hookworm*	33,69	31,24
63	m	12	*S. mansoni*	*T. trichuria*	32,05	30,48

Abbreviations: m = male, f = female, Ct = cycle threshold
