# Supplementary material for: Use of Occult Blood Detection Cards for Real-Time PCR-Based Diagnosis of Schistosoma Mansoni Infection
Source: PLoS One. 2015 Sep 11;10(9):e0137730. doi: 10.1371/journal.pone.0137730 (PMC4567332; doi:10.1371/journal.pone.0137730)
Supplement: S2 Table — (PDF) [file pone.0137730.s002.pdf]

S2 Table Dilutions of cloned IST-2 template:

N°	Plasmid specification	Dilution	Result 20.01.14		Result 21.01.14	
1	Schistosoma mansoni cloned ITS Standard*	10^1	positive	positive	positive	positive
2	Schistosoma mansoni cloned ITS Standard	10^2	positive	positive	positive	positive
3	Schistosoma mansoni cloned ITS Standard	10^3	positive	positive	positive	positive
4	Schistosoma mansoni cloned ITS Standard	10^4	positive	positive	positive	positive
5	Schistosoma mansoni cloned ITS Standard	10^5	positive	positive	positive	positive
6	Schistosoma mansoni cloned ITS Standard	10^6	positive	positive	positive	positive
7	Schistosoma mansoni cloned ITS Standard	10^7	positive	positive	positive	positive

*cloned by GenExpress™
